# FMF is not always “fever”: from clinical presentation to “treat to target”

**DOI:** 10.1186/s13052-019-0766-z

**Published:** 2020-01-15

**Authors:** Maria Cristina Maggio, Giovanni Corsello

**Affiliations:** Department of Health Promotion Sciences Maternal and Infantile Care, Internal Medicine and Medical Specialities “G. D’Alessandro”, University of Palermo, Palermo, Italy

**Keywords:** Familial Mediterranean fever, Autoinflammatory diseases, Colchicine, Canakinumab

## Abstract

Familial Mediterranean Fever, a monogenic autoinflammatory disease secondary to MEFV gene mutations in the chromosome 16p13, is characterized by recurrent self-limiting attacks of fever, arthritis, aphthous changes in lips and/or oral mucosa, erythema, serositis. It is caused by dysregulation of the inflammasome, a complex intracellular multiprotein structure, commanding the overproduction of interleukin 1. Familial Mediterranean Fever can be associated with other multifactorial autoinflammatory diseases, as vasculitis and Behçet disease.

Symptoms frequently start before 20 years of age and are characterized by a more severe phenotype in patients who begin earlier.

Attacks consist of fever, serositis, arthritis and high levels of inflammatory reactants: C-reactive protein, erythrocyte sedimentation rate, serum amyloid A associated with leucocytosis and neutrophilia. The symptom-free intervals are of different length.

The attacks of Familial Mediterranean Fever can have a trigger, as infections, stress, menses, exposure to cold, fat-rich food, drugs.

The diagnosis needs a clinical definition of the disease and a genetic confirmation. An accurate differential diagnosis is mandatory to exclude infective agents, autoimmune diseases, etc.

In many patients there is no genetic confirmation of the disease; furthermore, some subjects with the relieve of MEFV mutations, show a phenotype not in line with the diagnosis of Familial Mediterranean Fever. For these reasons, diagnostic criteria were developed, as Tel Hashomer Hospital criteria, the “Turkish FMF Paediatric criteria”, the “clinical classification criteria for autoinflammatory periodic fevers” formulated by PRINTO.

The goals of the treatment are: prevention of attacks recurrence, normalization of inflammatory markers, control of subclinical inflammation in attacks-free intervals and prevention of medium and long-term complications, as amyloidosis. Colchicine is the first step in the treatment; biological drugs are effective in non-responder patients.

The goal of this paper is to give a wide and broad review to general paediatricians on Familial Mediterranean Fever, with the relative diagnostic, clinical and therapeutic aspects.

## Background

Monogenic autoinflammatory diseases (AID) are a heterogeneous group of clinical conditions characterized by a dysregulation in innate immunity, secondary to mutations in genes involved in the regulation of inflammation. They are not associated to HLA antigens, gender, antigen-specific T lymphocytes and/or high-titer autoantibodies. Their signature is typically Interleukin (IL)-1 beta, overexpressed particularly in response to stressing and/or infective stimulus. However, in the last years a new group of AID have been described and these conditions are associated to increased secretion of interferon and can have associated autoimmune diseases [1]. Otherwise, year by year new clinical autoinflammatory conditions are described, increasing the connection between autoinflammation, autoimmunity and immunodeficiency.

A subset of these diseases, the hereditary recurrent fevers, includes Familial Mediterranean Fever (FMF), the tumor necrosis factor receptor-associated periodic syndrome (TRAPS), the Mevalonate Kinase Deficiency (MVK), and cryopyrin-associated periodic syndromes (CAPS). This paper focus on FMF, a monogenic AID, secondary to MEFV gene mutations in the chromosome 16p13, and typically characterized by recurrent self-limiting attacks of fever, arthritis, aphthous changes in lips and/or oral mucosa, erythema, serositis [1–3]. FMF is caused by dysregulation of the inflammasome, a complex intracellular multiprotein structure, commanding the overproduction of interleukin-1(IL-1). Moreover, FMF can be associated with other multifactorial autoinflammatory diseases, as vasculitis and Behçet disease [4].

The goal of this paper is to give a wide and broad review on Familial Mediterranean Fever, with the relative diagnostic, clinical and therapeutic aspects. Many studies have been performed on this topic especially in the last years, with new approaches for the treatment and the follow up. This paper offers to general paediatricians a guide in the complex world of Familial Mediterranean Fever and a rapid consultation to help young patients affected by this condition.

## Clinical presentation of FMF

Symptoms frequently start before 20 years of age. Clinical phenotype is usually more severe in patients with a precocious beginning of the attacks.

Patients display recurring clinical attacks consisting of fever, serositis, arthritis and high levels of inflammatory reactants: C-reactive protein (CRP), erythrocyte sedimentation rate (ESR), serum amyloid A (SAA) associated with leucocytosis and neutrophilia, with symptom-free intervals of different length.

The attacks of FMF can recognize a trigger in infections, stress, menses, exposure to cold, fat-rich food, some drugs.

**Fever** is a typical symptom in more than 96% of inflammatory attacks, with frequent high levels of body temperature, between 38 and 40 °C. 25% of the patients show chills before fever. The length of the self-limited episodes, lasting from 1 to 4 days, can have mild prodromal symptoms (myalgia, arthralgia, lumbar spine pain, headache, dyspnoea, nausea, arthralgia, asthenia, disquiet) ongoing for about 17 h.

Clinically, we can distinguish 3 phenotypes of FMF:

- type 1: acute attacks of fever plus the classical symptoms of painful serositis and arthritis;
- type 2: kidney amyloidosis, without other symptoms of FMF and without fever attacks;
- type 3: patients with two mutations of MEFV gene, without fever, other symptoms of FMF, nor amyloidosis.

The recurrence of attacks may differ: attacks frequency fluctuates from 1 per week to 1 per decade. Over the course of this lifetime disease, a patient can show different forms of attacks, however, most patients frequently experience the same symptoms at every attack.

Although in some reports a genotype-phenotype correlation is described, it is not strictly predictive and subjects with the same phenotype in the same family can show different symptoms.

Symptoms can also change in the course of life, and young children can have a different clinic presentation from adults.

However, patients’ age at the clinical symptoms is significantly associated with the clinical severity of the disease. Patients older than 12 years at the onset, showed a reduced frequency of fever attacks than the younger patients. Patients younger than 5 years at the onset showed more severe attacks [5].

Furthermore, occasional infections may show a more severe presentation with higher fever, in patients with MEFV mutations, for the amplification of cytokines secretion, especially of IL-1 beta.

**Abdominal pain** is a frequent symptom (90%) and may be diffuse or localized; in some cases, it typically mimics acute abdomen or appendicitis. 30–40% experiences unnecessary abdominal surgery. Acute peritonitis or intestinal obstruction can occur in these patients, in association with fever and/or other typical symptoms of FMF or as unique manifestation of the attacks. In some cases, anatomopathological study of appendix shows non-specific inflammation and laparoscopy and/or ultrasound reveal mesenteric lymphadenitis.

Some patients have diarrhoea and/or vomiting, whereas other patients experience constipation.

Functional gastrointestinal disorders are more frequent in children with FMF, and between those, functional dyspepsia and irritable bowel syndrome are more frequent [6].

In FMF patients with dyspeptic symptoms, endoscopic study reveals frequent pathological signs as chronic gastritis, esophagitis, signs of inflammatory bowel disease (IBD) by histopathological study of ileum and colon samples. Suggestive of IBD are goblet cell loss, cryptic hyperplasia, cryptitis.

The controversial role of MEFV mutations in developing Crohn disease is highlighted in study that demonstrated that IBD was more frequent in patients with FMF than in controls [7]. Amyloidosis and FMF attacks are more frequent in patients with the association of FMF and Crohn disease [8].

**Thoracic pain** is associated with pericarditis and/or pleural effusion (frequently monoliteral, of mild entity.

**Arthritis** is reported in 45% of FMF patients. It is frequently monoarticular, mainly involving main articular districts of lower limbs (hip, knee, ankle). Some patients experience arthralgia and do not manifest overt arthritis. Destruction of the hip was observed in some patients [9].

**Myalgia** is mostly localized at the legs, while some patients experience protracted febrile myalgia.

**Erysipelas-like erythema** is a typical clinical expression of FMF with a variable incidence (7–40%).

**Headache** is due to aseptic meningitis and, in association with asthenia, may be the main symptom [10].

In Turkey, abdominal pain (76%) and fever (58%) are the most frequent symptoms, followed by arthritis (28%), chest pain (19%) [10]. MEFV mutations were identified in 45%, whereas 55% had no identifiable mutations. 60% patients were heterozygous, 24.7% were compound heterozygous, 14% were homozygous, and the most frequent mutations were M694 V (48%), E148Q (18%), M680I (15%), V726A (12.5%), P369S (3.3%), R761H (0.9), K695R (0.9), E148V (0.9) and A744S (0.5%). Typical symptoms of FMF were more severe in patients with one or more M694 V or M680I mutations. E148Q or V726A mutations were associated with a mild phenotype. Fever associated with abdominal pain and chest pain was more frequent in patients with P369S, while arthritis was frequent in K695R heterozygous patients [10].

In a recent study performed in Japan, was reported that patients with MEFV exon 10 mutations showed earlier onset of the disease and frequently showed serositis, whereas the patients with the absence of MEFV exon 10 mutations showed later onset of FMF, with musculoskeletal manifestations as arthritis, myalgia, and erysipelas-like erythema [11]. This group if patients more frequently showed mutations in exon 3. There was no significant difference in the responsiveness to colchicine between the two groups. Furthermore, as demonstration of the difference in clinical phenotype depending on geographical area, Japanese patients affected by FMF show a more atypical phenotype compared to patients of areas were FMF is endemic [11]. The frequency of FMF patients with high-penetrance mutations, such as *MEFV* exon 10, is smaller in Japan than in Western countries.

In an Italian centre, the incidence of symptoms in more than 370 patients, was different [12]: fever (93.3%); abdominal pain (80.7%); arthralgia (66.9%); thoracic pain (40.2%); myalgia (36.3%); skin lesions (31.2%); aphthous lesions (28.2%); kidney involvement (15.4%); recurrent orchitis (3.5%).

Recently, cochlear involvement was demonstrated in FMF patients. Hearing thresholds show acute changes during the attacks, as an effect of acute inflammation on cochlea and recurrent inflammatory periods have a cumulative damage on cochlea. Furthermore, colchicine seems to improve hearing in these patients [13].

### Diagnostic criteria

The diagnosis of FMF needs a clinical definition of the disease, and a genetic confirmation.

An accurate differential diagnosis is mandatory, to exclude infective agents, autoimmune diseases, systemic Juvenile Idiopathic Arthritis, inflammatory bowel diseases [14–16] who can simulate the beginning of the attacks. However, the typical recurrent episodes can help physicians in the diagnosis.

Nevertheless, many patients have no genetic support, and in some subjects with the relieve of MEFV mutations, the phenotype is not in line with the diagnosis of FMF. For these reasons, diagnostic criteria for the diagnosis of FMF were developed in these years.

Tel Hashomer Hospital criteria were published starting from clinical observations in adult Israeli patients and are the most widely used for diagnosis of FMF. The diagnosis of FMF needs two major criteria or one major and two minor criteria (Table 1) [17].

**Table 1:** Tel Hashomer criteria

*Major criteria*	
1) Recurrent febrile episodes accompanied by peritonitis, synovitis, pleurisy	
2) AA amyloidosis without a predisposing disease	
3) Response to continuous colchicine administration	
*Minor criteria*	
1) Recurrent febrile episodes	
2) Erysipelas-like erythema	
3) FMF diagnosed in a first-degree relative	

More recently, the “Turkish FMF Paediatric criteria” were performed for children with the suspicion of FMF; these criteria were defined on a genetically group of FMF children, carrying two MEFV mutations [18]. The diagnosis requires the presence of 2 out of 5 criteria (table 2). However, these Yalcinkaya-Ozen criteria show a higher sensitivity, but a lower specificity than the Tel-Hashomer criteria. Ethnic origin and residence of the patients are not relevant on their sensitivity and specificity (Table 2) [19].

**Table 2:** Turkish FMF Paediatric criteria

Fever (axillary temperature > 38 °C)*	
Abdominal pain*	
Chest pain*	
Oligoarthritis*	
Family history of FMF	

*(6–72 h of duration, ≥3 attacks)

All these criteria do not include genetic study of MEFV. PRINTO promoted a study to develop and validate a set of clinical criteria for the classification of patients affected by inherited periodic fevers. This approach allowed identification of ‘positive’ and ‘negative’ criteria correlated with each disease. FMF diagnosis is supported by the presence of fever lasting less than 2 days, chest and/or abdominal pain, ethnicity. Otherwise, the absence of: fever lasting more than 6 days, enlarged cervical lymph nodes, urticarial rash, aphthous stomatitis supports the diagnosis [20].

The genetic study of FMF is a clue for diagnosis; laboratory tests can support the clinicians in the interpretation of the clinical picture. An acute attack is associated with increased CRP, ESR, SAA, immunoglobulins, neutrophil leucocytosis. However, these laboratory findings are not patognomonic.

## Treatment

The goals of the therapeutic approach in patients with FMF are to prevent the recurrence of attacks, to normalize inflammatory markers (CRP, ESR, SAA), to minimise subclinical inflammation in attacks-free intervals and to prevent the medium and long-term complications. In fact, increased SAA may lead to secondary amyloidosis and deposition of this insoluble protein in the kidney, heart, liver, gut, spleen, etc.

These objectives are correlated with the necessity to guarantee a good quality of life to paediatric patients and their family.

The first step in treatment of patients with recurrent attacks, even without fever, is colchicine [21].

Colchicine is effective also in children with a significant increase of SAA without fever and/or other symptoms linked to MEFV mutations.

The dosage is 0.5–1 mg/day and a therapeutic trial for at least 3–6 months is useful to establish the real response to the drug.

Treatment with colchicine should be started as soon as a clinical diagnosis is made.

A *starting* dose of ≤0.5 mg/day for children younger than 5 years of age, 0.5–1.0 mg/day for children 5–10 years of age, 1.0–1.5 mg/day for children > 10 years of age and in adults is recommended by the EULAR recommendations for the management of FMF [22]. In patients with pre-existing amyloidosis or high disease severity, higher doses may be prescribed. Colchicine side effects as abdominal pain, vomiting, diarrhoea in some cases may be reduced by dietary restrictions, dividing the dose in two times/day and/or a temporary reduction of the dose.

If inflammation persists despite a good adherence to the treatment, the dose may be gradually increased up to 2 mg/day in children and 3 mg/day in adults, carefully monitoring side effects.

Disease severity and patients’ tolerance to the recurrence of the symptoms are necessary elements guiding the physician in the obtainment of a personalized colchicine dose.

Colchicine is less effective in the control of myalgia and arthritis, requiring adding non-steroidal anti-inflammatory drugs or corticosteroids. In the few patients resistant to colchicine, other measures, including corticosteroids, are used in many centers, expecially on demand and in correlation with the severity of the attacks.

Patients who have one or more attacks/month despite receiving the higher tolerated dosage for 6 months or more, may be considered non-responder or resistant to colchicine, and must receive the anti-IL-1 beta biological drug, Canakinumab, as recently documented [23].

## Conclusions

The goal of the treatment in AIDs is the remission of the symptoms, the prevention of complications, the normalization of biochemical parameters and a good quality of life. For these reasons, treatment must be started as soon as possible, drugs need to be targeted to the single patient [24] and the therapeutic choose needs the cooperation between the clinicians, the patients and their families. Children need to be considered in the treatment decisions. In fact, these therapies may be long lasting and frequently need to be considered till adult age or lifelong.

The safety profile of Canakinumab is widely documented in paediatric patients [24] and the quality of life improves significantly with a treatment administered monthly with sub cutaneous injections.

## Data Availability

Materials and data of the patient are included in the medical records of the patient.

## Abbreviations

AID: Monogenic autoinflammatory diseases
CAPS: cryopyrin-associated periodic syndromes
CRP: C-reactive protein
EBV: Epstein-Barr virus
ESR: Erythrocyte sedimentation rate
FMF: Familial Mediterranean Fever
IBD: Inflammatory bowel disease
IL-1: Interleukin-1
MVK: Mevalonate kinase deficiency
SAA: Serum amyloid A
TRAPS: Tumor necrosis factor receptor-associated periodic syndrome
